# Balancing detectivity and sensitivity of plasmonic sensors with surface lattice resonance

**DOI:** 10.1515/nanoph-2023-0225

**Published:** 2023-09-07

**Authors:** Zhichao Li, Ciril S. Prasad, Xielin Wang, Ding Zhang, Rosemary Lach, Gururaj V. Naik

**Affiliations:** Department of Electrical and Computer Engineering, Rice University, Houston, TX 77005, USA; Applied Physics Program, Rice University, Houston, TX 77005, USA; Department of Physics and Astronomy, Rice University, Houston, TX 77005, USA

**Keywords:** biosensors, plasmonics, metasurface

## Abstract

Resonators are at the core of optical sensors enhancing light–analyte interaction and leading to higher sensitivities. Maximizing the sensitivity is an obvious objective function for the resonator design. However, high sensitivity does not guarantee sufficient detectivity. When the optical energy budget is limited, as in sensors on mobile platforms, a higher sensitivity usually leads to lower detectivity for nanophotonic sensors. In such scenarios, resonator design requires balancing the trade-off between the sensitivity and detectivity of the resonant sensor. Here, we show the direct dependence of detectivity on the *Q*-factor and the trade-off between the *Q*-factor and sensitivity. We study this trade-off in an array of plasmonic resonators. We choose plasmonic resonators because of their high sensitivity arising from large local field enhancements. Then, we show that the detectivity of this sensor may be boosted for limited energy budget applications by making an array of resonators supporting a surface lattice resonance (SLR). We experimentally demonstrate sensing and detection of antimouse IgG protein in a gold nanodisk array–based SLR sensor for various energy budgets.

## 1 Introduction

Label-free detection of biomolecules using light is becoming more popular, especially on mobile platforms where the optical energy budget is limited [1–3]. The performance of such optical sensors is captured by two important parameters, sensitivity and detectivity. The sensitivity is the shift in resonance peak wavelength with the analyte concentration. It depends on the local intensity of light interacting with the analyte molecules. On the other hand, detectivity captures the ability of the sensing system to accurately measure the concentration of the analyte in the presence of noise [4]. Detectivity is the most important parameter in energy-constrained sensing systems.

In resonant optical sensors, detectivity depends on the sensitivity and the *Q*-factor of the resonant mode [5]. For the same optical energy budget, detector noise, and sensitivity, a resonator with higher *Q*-factor results in higher detectivity. Similarly, when everything else is constant, higher sensitivity results in higher detectivity. Thus, high detectivity requires both high sensitivity and a high *Q*-factor [6, 7]. However, it turns out that the sensitivity and *Q*-factor trade-off in nanophotonic designs [8].

**Figure 1:**
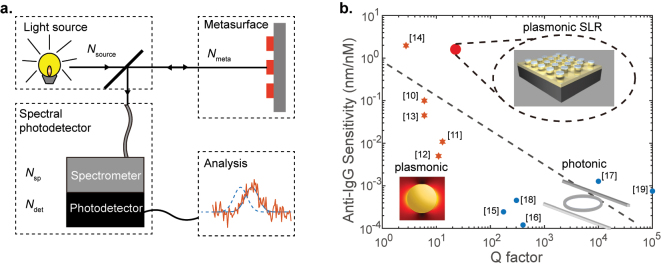
Detectivity and sensitivity of nanophotonic sensors: (a) the sketch of an optical sensor system. It contains the light source, the metasurface chip, the photodetector, and the analysis part. Each part of the system will induce some noise to the final result. (b) The trade-off between the *Q*-factor and the sensitivity of anti-IgG sensors. The data presented are collected from the literature [10–19]. The orange stars and blue circles correspond to plasmonic and photonic sensors, respectively. The red dot shows the result from our plasmonic surface lattice resonance design.

To understand the trade-off effect, consider a typical sensing system shown in Figure 1a. The sensing system consists of a light source of fixed power, a metasurface sensing element, a spectro-photodetector, and the postprocessing of the raw data carried out on a computer (for extracting the resonance peak shift and such other information). The raw data are acquired by integrating the optical signal at the detector for a chosen time period. The optical energy budget is thus the product of input optical power from the source and the detector integration time.

Each part of the sensing system contributes its own noise to the final result. The noise from the metasurface *N*_meta_ will be amplified by the sensitivity S of the sensor, and then add up to the light source *N*_source_, the spectrometer noise *N*_sp_, and the photodetector noise *N*_det_. Some examples of noise from the metasurface could be nonuniform analyte interaction, photothermal effects from the illumination, and nonlinear effects. Finally, the raw data containing the noise from all the components in the optical path transforms in the data processing unit before giving out the final sensing result. In this work, we assume that all the noise sources are additive white Gaussian sources and the raw data analysis is a Gaussian fit routine to find the resonance peak.

For a fixed energy budget *E* (product of incident intensity and data acquisition time), the signal-to-noise ratio (SNR) of the raw data output from the photodetector will be

(1) $$\begin{array}{ll} {SNR}_{\text{rawdata}} & =\frac{E}{SkN_{\text{meta}}+N_{\text{source}}+N_{\text{sp}}+N_{\det}}\\ & =\frac{E}{\left(Sk\alpha +\beta \right)N_{\det}} \end{array}$$

Here *k* converts the noise to the equivalent refractive index change since the sensitivity S is defined as the shift in peak wavelength per unit change in the local refractive index of the metasurface. For simplifying the equation above, we define $\alpha =\frac{N_{\text{meta}}}{N_{\det}}$ , $\beta =\frac{N_{\text{source}}+N_{\text{sp}}+N_{\det}}{N_{\det}}$ .

Using the error analysis for a least mean square Gaussian peak fitting procedure to find the resonance peak [9], the SNR in the extracted peak shift *δλ* will be

(2) $${SNR}_{\delta \lambda }=C\frac{S\;\Delta n}{FWHM}\sqrt{\frac{FWHM}{d\lambda }}{SNR}_{\text{rawdata}}$$

where Δ*n* is the change in the effective refractive index of the analyte layer, FWHM is the full width-half maximum of the resonance, *dλ* is the spectral resolution of the spectrophotometer, and *C* is a proportionality constant.

To fully capture the peak, the spectral resolution of the spectrophotometer *dλ* should be smaller than the peak FWHM. To capture the peak of a higher *Q*-factor, a smaller spectral resolution is required. For a fair comparison of optical sensors with different *Q*-factor, we set the ratio between the peak FWHM and the resolution *dλ* to be a constant *m* such that *mdλ* = FWHM. Using this information and substituting Eq. (1) for SNR_rawdata_ in Eq. (2), we get the overall SNR of the optical sensing system, given by Eq. (3).

(3) $${SNR}_{\delta \lambda }=\frac{C}{\lambda_{0}}\sqrt{m}\frac{\Delta nSQ}{Sk\alpha +\beta }\frac{E}{N_{\det}}$$

where *λ*_0_ is the peak wavelength of the sensor with no analyte. Using the definition of specific detectivity $D_{\det}^{*}$ of a detector (the reciprocal of noise equivalent power (NEP) per unit area of the detector), the overall specific detectivity (D*) of the optical sensing system is given by Eq. (4).

(4) $$D^{*}=\frac{C^{\prime }}{\lambda_{0}}\sqrt{m}\frac{SQ}{Sk\alpha +\beta }D_{\det}^{*}$$

where *C*′ is a proportionality constant. From Eq. (4), we know that in the low-sensitivity regime, increasing sensitivity will increase the detectivity of the sensor, but when sensitivity is large, the Skα term in the denominator of Eq. (4) can’t be ignored. Thus, increasing sensitivity alone is not sufficient for good detectivity. However, maximizing the product of S and *Q* directly helps with detectivity.

In nanophotonic sensors, the sensitivity and *Q*-factor of a resonator are related to each other. Barton et al. [8] identified that the sensitivity and *Q*-factor of nanophotonic sensors do trade-off. Qualitatively, the origin of this trade-off may be understood as follows: the sensitivity of the optical sensor is related to the near field [20, 21]. When fields are strongly localized, their far-field radiation loss, governed by the Fourier dual of field distribution, is high. Thus, the *Q*-factor of the resonator is low. Thus, there exists a trade-off between *Q*-factor and local field enhancement in simple nanophotonic resonators.

By comparing a series of anti-IgG protein sensors reported in the literature, and plotting their sensitivity and *Q*-factor in Figure 1b, the trade-off between the *Q*-factor and sensitivity is evident. We chose to focus on anti-IgG sensors due to the importance of IgG and anti-IgG proteins in pathology. Immunoglobulin G (IgG) is the most common antibody in human blood and is responsible for immunity against many viruses, bacteria, and fungi [22]. IgG binds specifically to anti-IgG, and often the detection of IgG and anti-IgG is both important in pathology.

In Figure 1b, at the high-sensitivity end, the sensors are mainly plasmonic [10], [11], [12], [13, 23, 24]. And at the high *Q*-factor end, the sensors are mainly photonic [16, 19, 25, 26]. Among photonic sensors, resonators based on whispering gallery modes can reach *Q*-factors of 10^5^ [27, 28]. The trend line suggests $S\propto Q^{-0.5}$ . With this trend substituted in Eq. (4), high detectivity demands a high *Q*-factor, not high sensitivity from the sensor.

Detectivity becomes the performance-limiting parameter when the energy budget of the sensor is limited. When *E* is much bigger than all the combined strengths of the noise sources, the sensor can afford high sensitivity at the cost of some detectivity. In such cases, plasmonic sensors are the best. However, when *E* is comparable to or only slightly better than the strengths of noise sources combined, a high *Q* photonic design is the optimum one. For any scenario in between these two extremes, an optimum design requires a plasmonic–photonic hybrid.

There are many methods reported in the literature to achieve hybrid plasmonic–photonic designs. However, plasmonic surface lattice resonance (SLR)-based design offers flexibility and performance. A plasmonic SLR is achieved by an array of plasmonic resonators that significantly narrow down the plasmonic resonance peak [29]. Recent works have shown that such designs can achieve ultra-high *Q* factors in plasmonic metasurfaces [30].

Here, we employ an array of plasmonic resonators supporting an SLR as an antimouse IgG sensor to demonstrate its high detectivity and high sensitivity. We demonstrate the detection of antimouse IgG protein using this plasmonic SLR sensor for various energy budgets.

## 2 Results and discussion

**Figure 2:**
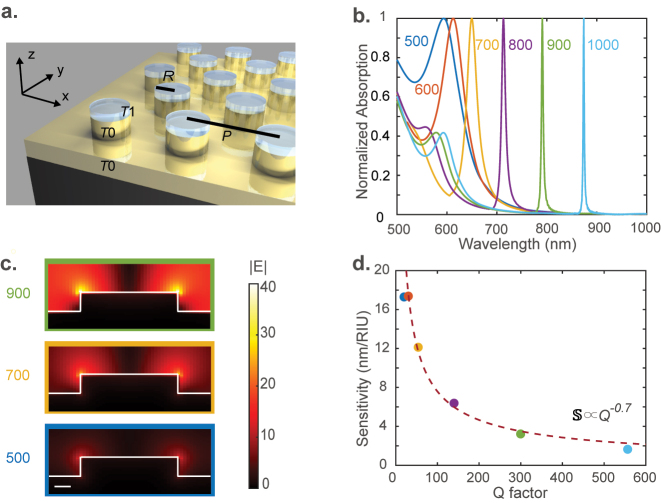
Design of plasmonic SLR resonators: (a) the schematic of the plasmonic SLR array comprising a hexagonal lattice of gold cylinders with a period *P* on a *T*_0_ = 60 nm thick layer of gold deposited onto a glass substrate. The radius of the gold cylinder is *R* = 150 nm. The analyte is modeled as a *T*_1_ = 5 nm thick dielectric layer of index varying from 1 to 1.2 on top of the cylinders. In simulations, we use an *x*-polarized (TM, *y*-polarized wave is TE) plane wave light source propagating along the minus *z*-axis. (b) The calculated absorption spectra of the structure with different periods *P* are indicated in the figure in nm. (c) Field distribution in a cross section of the structure at the resonance peak wavelength for three chosen values of *P* = 500, 700, and 900 nm. The color of the bounding box corresponds to that of the absorption curve in (b). The scale bar represents 50 nm. (d) The simulated refractive index sensitivity of our metasurface device in the units of nm shift in resonance peak per refractive index unit (RIU) plotted against its *Q*-factor. The colors of the dots correspond to the colors of the curves in panel (b). The dashed line is the best-fit curve of the form $SQ^{\alpha }=$ constant. Here, *α* = 0.7049.

Our sensing device comprises a hexagonal array of 60 nm tall gold cylinders on top of a 60 nm thick gold film deposited onto a substrate. The gold cylinders have a radius of 150 nm. The distance between the two closest cylinders or the period *P* ranges from 500 to 1000 nm in this study. The schematic of the sensor is shown in Figure 2a. Using Lumerical finite-difference time-domain (FDTD), we simulate the design under a normal incident plane wave light source. We model the analyte interaction in simulations by a 5 nm thick dielectric layer on top of the gold cylinders. The index of this dielectric layer is varied from 1 to 1.2 to capture the effect of analyte concentrations in the range of 0–8 nM.

The simulated absorption spectra of the device are shown in Figure 2b for different periods *P*. A larger period redshifts the resonant peak and also makes it sharp. The *Q*-factor of the mode increases with the period because the resonance moves deeper into the bandgap of the array, which arrests the radiative loss. The highest *Q*-factor observed for *P* = 1 µm is about 560. The field distribution in a cross section of the resonator at resonance is shown in Figure 2c for three different values of *P*. The highest peak local field occurs in the case of the smallest period of 500 nm. As the period increases, the *Q*-factor increases, but the peak local field enhancement drops down. With increasing periods, the destructive interference better arrests the radiative loss but at the cost of field localization. Increasing period spreads the local field over a larger volume resulting in a smaller peak field enhancement.

An intuitive approach to understanding the trade-off between the *Q*-factor and peak near-field enhancement is as follows: the *Q*-factor of the resonator is limited by the radiative loss. The radiative loss depends on the *k*-space overlap of the resonant mode with the *k*_0_-sphere, where *k*_0_ is the magnitude of the wavevector of light in free space. The *k*-space spread of the resonant mode is the Fourier dual of its spread in real space. Thus, higher localization of the mode results in larger radiative loss and hence smaller *Q*-factors. Thus, higher peak local field strength results in a smaller *Q*-factor.

Since higher maximum local field strength corresponds to stronger light–analyte interaction or higher sensitivity, the sensitivity and *Q*-factor trade-off. Figure 2d plots the sensitivity calculated from multiple simulations versus the *Q*-factor for the six periods chosen in Figure 2b. As expected from the argument presented in the previous paragraph, the sensitivity S trades off with the *Q*-factor approximately as $S^{2}Q=$ constant.

**Figure 3:**
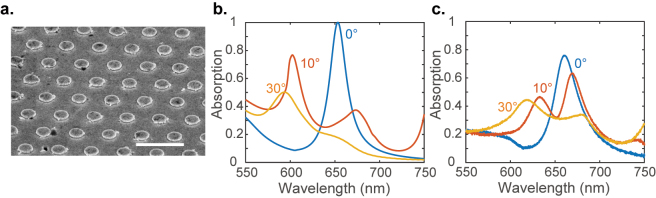
Plasmonic SLR device: (a) an SEM image of the as-plasmonic SLR structure with a period *P* = 700 nm. The scale bar represents 1 µm. The absorption spectra of the device for various incident angles were obtained from (b) simulations and (c) experiments.

Using the trade-off relationship between S and *Q* in Eq. (4), the detectivity scales as $\sqrt{Q}$ . To demonstrate improved detectivity in an array plasmonic sensor, we choose to fabricate the structure shown in Figure 2a with *P* = 700 nm. Though longer period structures promise even higher detectivity, we chose the structure with a period of 700 nm due to the limitation of our optical characterization setup. The longest wavelength that our optical characterization setup could reliably handle is 700 nm. The scanning electron microscope image of the sample is shown in Figure 3a. We use an angle-resolved microscope-based spectrophotometer to measure the reflectance spectra of our sample. The measurement setup used here is described in detail in the Methods section. The illumination is unpolarized in our experiments. We do not add a polarizer to our experimental setup because a linear polarizer cannot separate TM and TE polarizations in an objective-based setup. An objective is needed to characterize our sample because of its small area of nanopatterning. The simulated TM polarization and measured unpolarized absorption spectra of the device at various angles of incidence are shown in Figure 3b and c, respectively. The measured *Q*-factor of the resonance is about 20, which is higher than that for typical LSPR (localized surface plasmon resonance) resonators. The measured and the simulated spectra match only qualitatively. The differences arise from sample imperfections and the presence of the TE polarization in experiment. However, a qualitative agreement between the two may be noticed.

**Figure 4:**
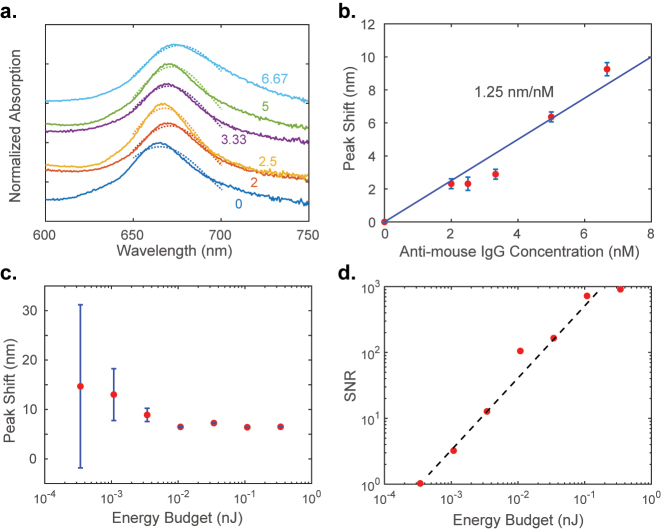
Measured sensitivity and SNR: (a) measured absorption spectra (waterfall plot with an offset of 0.3) of the plasmonic SLR subjected to analyte antimouse IgG of different concentrations as labeled in the units of nM. The dashed lines show the Gaussian fitting results. (b) The shift in the resonance peak wavelength with the analyte concentration. The slope of the fitted line, the sensitivity of the device, is 1.25 nm/nM. The error bars are one single measurement’s Gaussian fitting error. (c) The resonant wavelength shift evaluated from the Gaussian fits for the measured spectra at various incident optical energy budgets. The error bars are the standard deviations obtained from experiments and the peak fitting routine. The concentration of the analyte was 5 nM for all the measurements here. (d) The signal-to-noise ratio is calculated from the error bars of (c) versus the optical energy budget. The trend line shows the expected linear dependence with a slope of 2.74 per pJ.

The plasmonic SLR device was then tested for its ability to sense antimouse IgG protein. The device was functionalized and exposed to antimouse IgG solutions of different concentrations as described in the Methods section. This procedure was adopted from reference [31]. Then, the absorption spectra were collected for each concentration (see Figure 4a). The resonance peak wavelength was extracted by fitting the measured spectrum with a Gaussian curve. The shift in resonance peak (*δλ*) from that of the device with no exposure to antimouse IgG is plotted as a function of the concentration of the protein in Figure 4b. The sensitivity of the sensor to the protein is about 1.25 nm/nM. This sensitivity value is comparable to that of plasmonic sensors reported earlier [14].

To characterize the detectivity of this sensor, we fixed the concentration of the analyte and varied the optical energy budget. We held all the characterization parameters constant while changing only the illumination light intensity using a set of neutral density (ND) filters. We acquired the absorption spectrum on the sample 10 times for each ND filter setting. Then, running Gaussian fits toward the absorption peaks on each absorption data, we estimated the standard deviation or error in the peak shift estimation. Figure 4c plots the resonance peak shift for 5 nM analyte concentration estimated at various incident optical energy budgets. The energy budget for each spectrum acquisition was obtained by measuring the incident optical power at the detector in the spectral range of 600–750 nm and multiplying it with the integration time of the detector. The error bars in Figure 4c represent the standard deviation for each measurement set. The peak shift data are less reliable at the lower end of the illumination energy budget as can be noticed from the large error bars. The SNR of the sensor cannot be accurately estimated in this region. Figure 4d plots the SNR of the sensor as a function of the energy budget. With the increase of energy budget, the SNR increases correspondingly, and the trend line shows that the SNR is linearly proportional to the energy budget. The detectable SNR of unity occurs at the knee of the plot and continues to grow linearly with the energy budget at the rate of 2.74 per pJ. Thus, the detectivity of the sensor is 2.74 per pJ.

## 3 Conclusions

Maximizing the sensitivity is not a sufficient consideration in the design of a nanophotonic sensor. When the available optical energy budget is limited, detectivity must be considered as well. Here, we derived an expression relating the detectivity of a resonant optical sensor to its *Q*-factor and sensitivity. We showed that a higher *Q*-factor ensures higher detectivity or a lower minimum energy budget. However, the higher *Q*-factor results in smaller local field enhancements or sensitivity in plasmonic and many other single resonator photonic designs. We discussed the physics behind this trade-off and proposed an SLR-based design to build plasmonic sensors with better detectivity for limited energy budget applications. We demonstrated a plasmonic SLR sensor for sensing anti-mouse IgG protein and measured its sensitivity, *Q*-factor, and detectivity. This work highlighted the importance of understanding the trade-off between sensitivity and *Q*-factor in designing nanophotonic sensors. Though we considered only single resonator designs, the theory developed here could be extended to more complex resonator configurations where the trade-off between *Q*-factor and sensitivity may be relaxed to enable nanophotonic detectors with both high sensitivity and high detectivity.

## 4 Methods

### 4.1 Simulations

Full-wave electromagnetic simulations were performed using a commercial finite-difference time-domain solver (Lumerical). Si and Au optical constants were obtained from Palik [32] and fitted with Drude–Lorentz models. The simulations were carried out on a single period of the array with periodic boundary conditions. The light source was a plane wave source set 600 nm above the top of the metasurface structure. The frequency-domain field and power monitor planes were set 100 nm above the light source. Both the light source and the monitor were set to cover the whole simulation region. The mesh was uniform mesh with *x*, *y*, and *z* grid spacing of 10 nm, 10 nm, and 5 nm, respectively.

### 4.2 Sample fabrication

Planar nanofabrication was used for fabricating the metasurface. At first, we used e-beam evaporation to deposit a 60 nm thick gold layer on the Si substrate. Next, we carried out e-beam lithography (Elionix ELS-G100) to create a periodic hole pattern on the resist. Then, we evaporate 60 nm thick gold and liftoff to fabricate the metasurface. The fabricated devices were 0.5 mm × 0.5 mm in size.

### 4.3 Functionalization and biosensing

We use a 0.1 M solution of 8-mercaptooctanoic acid (8-MOA) from Sigma Aldrich in ethanol to functionalize our plasmonic SLR device. We let the sample sit in this solution for 12 h at 4 °C. Then we soak the sample in 2-(*N*-morpholino)ethanesulfonic acid (MES) buffer at a pH of 6.5 for 35 min. The MES buffer contained 0.4 M EDC or 1-ethyl-3-(3-dimethylaminopropyl)carbodiimide hydrochloride (Thermo Fisher) and 0.1 M NHS or *N*-hydroxysuccinimide (Sigma Aldrich). After drying the sample, we incubate the device with 100 μg/mL anti-CD63 antibodies (Ancell, 215-820) for 1 h at room temperature. The surfaces were subsequently blocked with 5 % bovine serum albumin or BSA (Sigma Aldrich, A8531) in phosphate buffer solution or PBS (Thermo Fisher, 10010023) for 30 min at room temperature. After rinsing with PBS, we immerse our device in anti-mouse IgG (Sigma Aldrich, B7264) of a particular concentration at 4 °C for 12 h. The original antimouse IgG solution is diluted with PBS buffer to solutions of concentrations 2 nM, 2.5 nM, 3.33 nM, 5 nM, and 6.67 nM. Then, after drying, the sample is subjected to optical characterization. After characterization, the same device is reused for sensing a different concentration of antimouse IgG. Before reusing, the device is rinsed to clean off all the added chemicals using SC-1 or a combination of ammonium hydroxide, hydrogen peroxide, and water in a volume ratio of 1:1:5 at room temperature for 90 min. The device is then thoroughly rinsed in DI water before reusing.

### 4.4 Spectra measurement

We use a Fourier-space imaging or energy-momentum imaging setup for Figure 3c. This setup allows for single-shot measurement of angle-dependent reflection (*R*) and transmittance (*T*) spectra on small-area samples. Absorption (*A*) is calculated as *A* = 1 − *T* − *R*. Inserting a Bertrand lens to a standard imaging spectrophotometer allows projecting the Fourier space onto the imaging device. More details of the setup may be found in our previous work [33]. Spectra in Figure 4 are measured using a spectrometer (AvaSpec-ULs2048L).

### 4.5 Spectra normalization

The normalization of the spectra in Figures 1b and 4a is carried out in such a way that the peak value of the curves remains at unity. The normalization of the spectra in Figure 3b is carried out by dividing each spectrum by the value of highest absorption in the 0° curve.

### 4.6 Optical energy budget

The optical energy budget for measurement is obtained by multiplying the integration time of the detector (2.5 s) with the optical power at the detector when the sample is replaced by a mirror. This power is extracted from the total photocurrent recorded by the power meter (ThorLabs, S121C). The spectral shape of the incident light from the quartz halogen bulb (Nikon, 12 V, 100 W, 2000 h), and the transmittance spectra of the short-pass and long-pass filters (Thorlabs FES0750, FESL0600) are used in the estimation of the optical power.
